# A novel function of NLRP3 independent of inflammasome as a key transcription factor of IL-33 in epithelial cells of atopic dermatitis

**DOI:** 10.1038/s41419-021-04159-9

**Published:** 2021-09-24

**Authors:** Jie Zheng, Lu Yao, Yijing Zhou, Xiaoqun Gu, Can Wang, Kaifan Bao, Yang Sun, Min Hong

**Affiliations:** 1 Jiangsu Key Laboratory for Pharmacology and Safety Evaluation of Chinese Materia Medica, School of Pharmacy, Nanjing University of Chinese Medicine, 138 Xianlin Avenue, Nanjing, 210023 China; 2 Department of Pharmacology, School of Medicine & Holistic Integrative Medicine, Nanjing University of Chinese Medicine, 138 Xianlin Avenue, Nanjing, 210023 China; 3 Department of Biotherapy, Nanjing Jinling Hospital, Nanjing, 210002 China; 4 Department of Immunology, School of Medicine & Holistic Integrative Medicine, Nanjing University of Chinese Medicine, 138 Xianlin Avenue, Nanjing, 210023 China; 5 State Key Laboratory of Pharmaceutical Biotechnology, Chemistry and Biomedicine Innovation Center, School of Life Sciences, Nanjing University, 163 Xianlin Avenue, Nanjing, 210023 China

**Keywords:** Mechanisms of disease, Inflammation

## Abstract

Atopic dermatitis (AD) is a common chronic pruritic inflammatory skin disorder characterized by recurrent eczematous lesions. Interleukin (IL)−33, a cytokine of the IL-1 family, was found to play an important role in the pathogenesis of AD. As a key component of the inflammasome, NLRP3 has been mostly described in myeloid cells that to mediate inflammasome activation conducted proinflammatory cytokine production of the IL-1 family. However, the role of NLRP3 inflammasome in the pathogenesis of AD, as well as IL-33 processing are highly controversial. Whether NLRP3 can mediate IL-33 expression and secretion independently of the inflammasome in the epithelium of AD has remained unclear. In this article, we found the mRNA expression of *Il33* and *Nlrp3* were notably increased in the lesional skin of AD patients compared to healthy controls. We then found a significant positive correlation between the expression of *Nlrp3* and *Il33* in the epithelium of MC903-mediated AD mice model, but no changes were observed for *Il36α*, *Il36γ*, *Il1β*, or *Il18* mRNA expression, as well as IL-1β or IL-18 production. Overexpression of NLRP3 in human immortalized epithelial cells increased IL-33 expression, whereas siRNA targeting NLRP3 abolished IL-33 expression. In addition, inhibition of NLRP3 inflammasome activation or caspase-1 activity with MCC950 or VX-765 showed no effect on the expression and secretion of IL-33 in AD mice. Unlike myeloid cells, NLRP3 predominantly located in the nucleus of epithelial cells, which could directly bind to *Il33* specific-promoters and transactivate it through an interaction with transcription factor IRF4. Furthermore, NLRP3 deficient mice exhibited a significant alleviated epidermis inflammation and decreased mRNA expression and secretion of IL-33 in MC903-mediated AD mice without interfering with TSLP and IL-1β production. Our results demonstrate a novel ability of NLRP3 to function as a crucial transcription factor of IL-33 in epithelium independently of inflammasome that to mediate the pathological process of AD.

## Introduction

Atopic dermatitis (AD) is a common chronic pruritic inflammatory skin disorder characterized by recurrent eczematous lesions. Interleukin (IL)−33, a cytokine of the IL-1 family, was found to play an important role in the pathogenesis of allergic diseases, i.e., AD [1, 2], asthma [3], allergic rhinitis [4], urticaria [5], and allergic conjunctivitis [6], and targeting the IL-33 inflammatory axis has therapeutic potential in controlling AD [7]. IL-33 is normally released by damaged or stressed endothelial or epithelial cells [8] that can induce type 2 cytokines mediated pathology at barrier surfaces [9]. Although full-length IL-33 (30 kDa) is originally identified as an inactive precursor, it has been proposed that full-length IL-33 is biologically active and passively released to extracellular spaces to mediate acute local inflammation. However, whether it is necessarily processed by caspase-1 is controversial [10–13]. Moreover, the molecular mechanism about the regulation of IL-33 gene expression and cytokine secretion has remained unclear [14].

NLRP3 is the best characterized cytosolic NOD-like pattern recognition receptor, which can detect a broad range of microbial motifs, endogenous danger signals, and environmental irritants. Activation of NLRP3 results in the assembly and activation of inflammasome with ASC and caspase-1 [15], which is required for cleavage and activation of caspase-1 that in turn cleaves pro- IL-1β and IL-18 to induce secretion of bioactive IL-1β and IL-18 [16]. As the key component of the inflammasome, NLRP3 has been mostly described in myeloid cells, in which it exhibits high expression. However, accumulating studies suggested NLRP3 is also expressed in lymphoid cells [17, 18], epithelial cells [19–21], or chondrocytes [22, 23]. Regardless of cell types, much current interest in NLRP3 has been still prompted by its role in the assembly and activation of inflammasome. Actually, NLRP3 itself was shown to affect a wide range of human diseases, highlighting the potential application of NLRP3-targeted therapies for these diseases [24].

It is worth noting that NLRP3 deficiency in macrophages attenuates IL-33 secretion, indicating NLRP3 is involved in IL-33 processing in myeloid cells [25]. Interestingly, a recent report demonstrated NLRP3 could regulate T helper (Th)2 program as a transcriptional regulator independently of inflammasome [26]. In addition, an inflammasome-independent effect of NLRP3 has also been reported in epithelial cells but the precise mechanism is not yet clear [27]. In this report, we suspected NLRP3 might regulate IL-33 expression and secretion in epithelial cells independently of inflammasome that to mediate the pathological process of AD.

## Results

### IL-33 expression and secretion correlates with NLRP3 expression in murine AD model

**Fig. 1:**
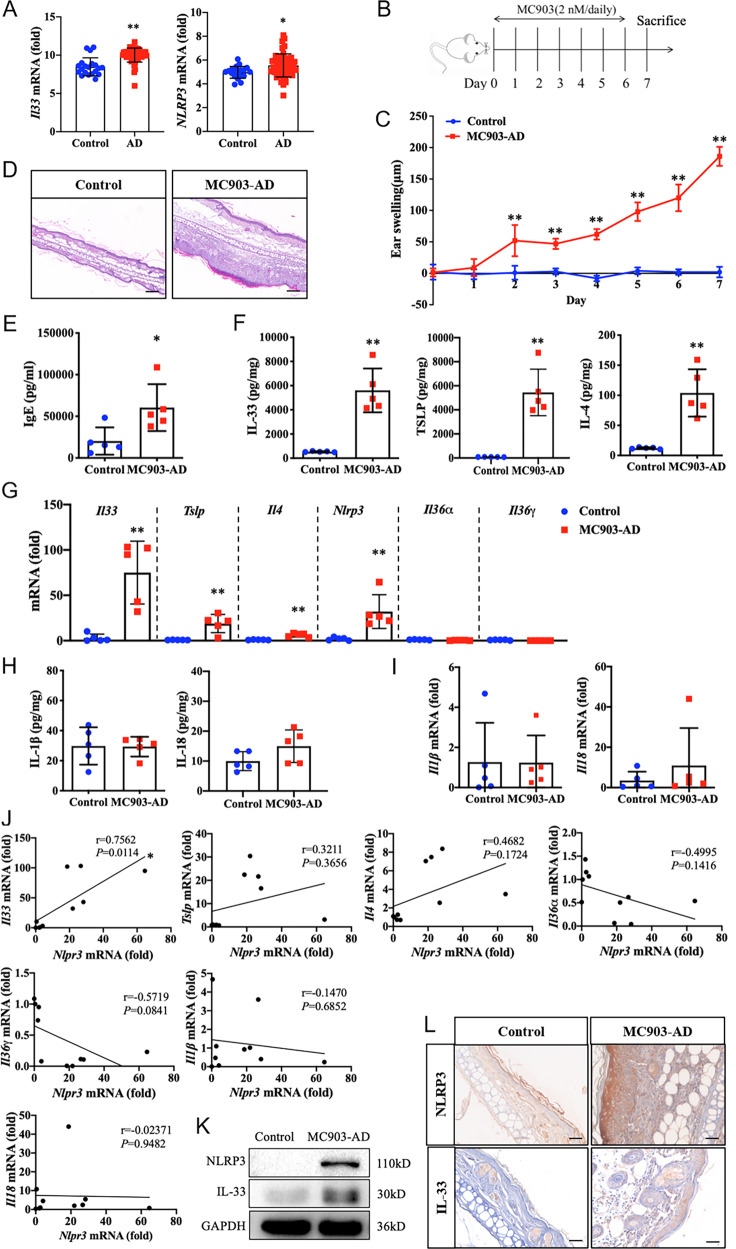
IL-33 expression and secretion correlates with NLRP3 expression in murine AD model. **A** The normalized expressions of genes encoding *Il33* and *Nlrp3* in skin tissues from healthy individuals (homo sapiens skin biopsy, Week 0, Normal, *n* = 20) and lesions from patients with atopic dermatitis (AD) (homo sapiens skin biopsy, Week 0, lesional, *n* = 51) were analyzed with the primary data obtained from the NCBI GEO dataset GSE130588. **B** Flow chats of MC903-induced AD mouse model. MC903 (2 nM, dissolved in 20 μL ethanol) was topically applied to the dorsal side of right ear in mice once a day from days 0 to 6 to induce AD model, control mice were applied with 20 μL ethanol on the dorsal side of right ear. **C** Ear thickness of mice was assessed daily by using a vernier caliper. Ear swelling (μm) was obtained by analysis of the thickness difference between the left and right ears of each mouse. **D** Ear histological changes were evaluated with H&E staining and images were observed with optical microscopy (magnification: ×200, scale bar: 100 μm). **E** Serum level of IgE was assessed by ELISA. **F** Cytokine secretion level for IL-33, TSLP, and IL-4 in mice ear homogenates was measured by ELISA. **G** Quantitative RT-PCR (qRT-PCR) analysis of *Il33, Tslp*, *Il4*, *Nlrp3*, *Il36α*, and *Il36γ* mRNA expression in mice ear homogenates. **H** Cytokine secretion level for IL-1β and IL-18 in mice ear homogenates was measured by ELISA. **I** qRT-PCR analysis of *Il1β* and *Il18* mRNA expression in mice ear homogenates. **J** Correlation analysis of the expression of *Nlrp3* mRNA and *Il33*, *Tslp*, *Il4*, *Il36α*, *Il36γ*, *Il1β*, and *Il18* mRNA in control and MC903-induced AD mice. **K** Immunoblot analysis of NLRP3 and IL-33 expression in mice ear homogenates. **L** Immunohistochemical (IHC) analysis of NLRP3 and IL-33 expression in mice ear homogenates (magnification: ×200, scale bar: 100 μm). Statistical comparisons were performed using unpaired two-tailed Student’s *t* test (all data are represented as the mean ± SD of three independent experiments, *n* = 5, *P < 0.05; **P < 0.01 vs. Control).

According to the primary data obtained from the NCBI GEO database (GSE130588), the normalized expressions of genes encoding *Il33* and *Nlrp3* in skin tissues from healthy individuals (*n* = 20) and lesions from patients with AD (*n* = 51) were analyzed. We found both *Il33* and *Nlrp3* mRNA expression were increased significantly in lesional skin from patients with AD compared with healthy controls (Fig. 1A). To further study the changes and functional effects of NLRP3 and IL-33 in AD, an MC903-induced murine AD model (Fig. 1B) was employed in the current study. MC903, a low-calcemic analog of vitamin D3, can promote a strictly IL-33 expression and induce changes in skin morphology and allergic inflammation resembling immune perturbation observed in acute lesions of AD patients [28]. We observed that topically applying with MC903 induced a gradually increased ear swelling (Fig. 1C) and more robust inflammatory infiltration of lymphocytes into ear epithelium (Fig. 1D), in line with notably elevated serum level of IgE (Fig. 1E) in AD mice than did in control. Moreover, cytokine secretion, as well as mRNA expression of IL-33, TSLP, and IL-4 increased remarkably in ear homogenates from AD lesions (Fig. 1F, G). Consistent with the GEO dataset analysis of patients with AD, the mRNA expression of *Nlrp3* was strictly enhanced in ear epithelium of AD mice (Fig. 1G). As the newly described members of the IL-1 family, IL-36α, and IL-36γ were reported to be upregulated to some extent in the lesional skin of AD patients [29]. However, the mRNA expression levels of *Il36α* and *Il36γ* did not appear to increase in MC903-induced AD mice (Fig. 1G). Furthermore, as the conventional members of the IL-1 family, the maturation and secretion of IL-1β and IL-18 are believed to be regulated by classic NLRP3 inflammasome signaling. However, no changes were detected in terms of the cytokine production of IL-1β and IL-18 (Fig. 1H), as well as mRNA expression of *Il1β* or *Il18* in ear homogenates of AD mice compared with control (Fig. 1I). Notably, we observed a significant positive correlation between the expression of *Il33* mRNA and that of *Nlrp3* mRNA, but not in the expression of *Tslp*, *Il4*, *Il36α*, *Il36γ*, *Il1β*, or *Il18* mRNA (Fig. 1J). In addition, the protein expression of NLRP3 increased obviously in line with IL-33 in ear homogenates from AD lesions by immunoblot analysis (Fig. 1K) and IHC staining (Fig. 1L). These results suggested IL-33 expression and secretion were positively correlated with NLRP3 expression in AD mice model but it might not be associated with classic NLRP3 inflammasome activation, as the production and mRNA expression of IL-1β or IL-18 were not affected.

### NLRP3 regulates IL-33 expression and secretion in epithelial cells

**Fig. 2:**
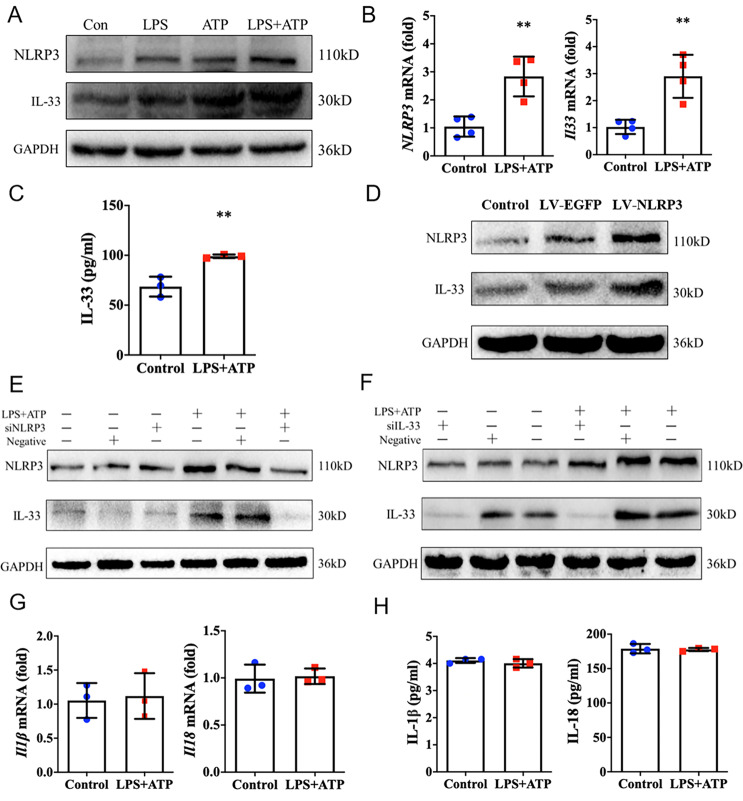
NLRP3 regulates IL-33 expression and secretion in epithelial cells. **A** Immunoblot analysis of NLRP3 and IL-33 protein expression in HaCaT cells in response to LPS (10 μg/mL, 24 h), ATP (5 mM, 1 h), or LPS (10 μg/mL, 24 h) and ATP (5 mM, 1 h) stimulation. **B** Relative expression of *Nlrp3* and *Il33* mRNA in HaCaT cells in response to LPS (10 μg/mL, 24 h) and ATP (5 mM, 1 h) stimulation by qRT-PCR analysis. **C** ELISA analysis of IL-33 secretion level from HaCaT cells in response to LPS (10 μg/mL, 24 h) and ATP (5 mM, 1 h) stimulation. **D–F**, Immunoblot analysis of NLRP3 and IL-33 protein expression in HaCaT cells following overexpression of NLRP3 by transfecting with pLV-EGFP-NLRP3, or interfering of NLRP3 or IL-33 with specific siRNAs. **G** qRT-PCR analysis of *Il1β* and *Il18* mRNA expression in HaCaT cells upon LPS (10 μg/mL, 24 h) and ATP (5 mM, 1 h) stimulation. **H** ELISA analysis of the secretion of IL-1β and IL-18 from HaCaT cells in response to LPS (10 μg/mL, 24 h) and ATP (5 mM, 1 h) stimulation. Statistical comparisons were performed using unpaired two-tailed Student’s *t* test (all data are represented as the mean ± SD of three independent experiments, *P < 0.05; **P < 0.01 vs. Control).

We next investigated whether NLRP3 can mediate IL-33 expression and secretion in epithelial cells ex vivo. Human immortalized keratinocytes (HaCaT) were utilized with stimulation of LPS and extracellular ATP, the classic activators of NLRP3. We found more robust protein expression of NLRP3 and IL-33 in HaCaT cells in response to stimulation compared to control (Fig. 2A). Increased protein expression of NLRP3 and IL-33 were also detected in 16HBE cells (human bronchial epithelial cells) in response to stimulation (Supplementary Fig. 1A). Consistently, mRNA expression of *Nlrp3* and *Il33* in HaCaT cells notably upregulated in response to stimulation (Fig. 2B), and cytokine secretion of IL-33 significantly elevated (Fig. 2C). Moreover, NLRP3 overexpression in HaCaT cells induced an evident increase expression of IL-33 (Fig. 2D), whereas NLRP3 deficiency decreased IL-33 expression both in HaCaT cells (Fig. 2E) and 16HBE cells (Supplementary Fig. 1B). To the contrary, IL-33 deficiency did not alter the expression of NLRP3 in both HaCaT cells (Fig. 2F) and 16HBE cells (Supplementary Fig. 1C). As observed in vivo, we noted that stimulation with LPS and ATP, failed to induce the mRNA expression and cytokine secretion of IL-1β and IL-18 in HaCaT cells (Fig. 2G, H). These results demonstrated that NLRP3 was able to regulate IL-33 expression and secretion in epithelial cells, but failed to induce IL-1β and IL-18 production ex vivo.

### NLRP3 regulates IL-33 expression independently of inflammasome in epithelium

**Fig. 3:**
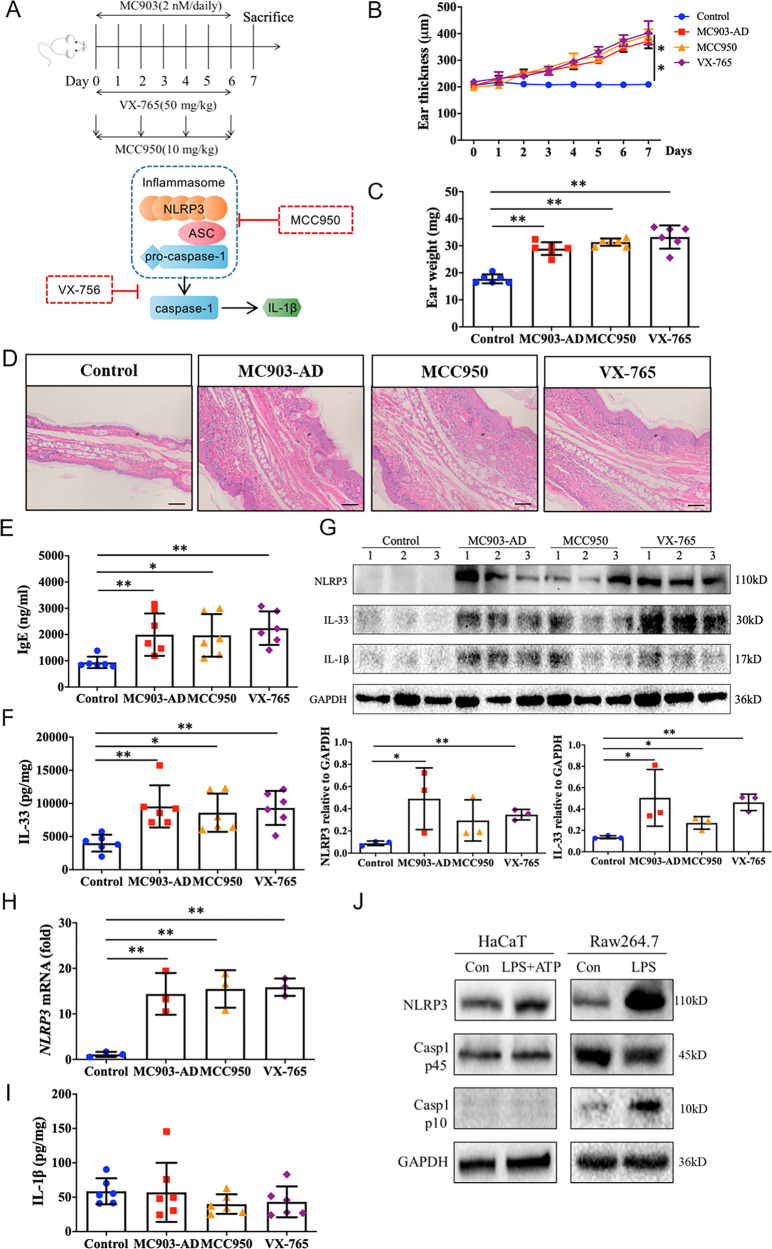
NLRP3 regulates IL-33 expression independently of inflammasome activation in epithelium. **A** Flow chats of MC903-induced AD mouse model, as well as the dosage regimen and targets of MCC950 or VX-756. MC903 (2 nM, dissolved in 20 μL ethanol) was topically applied to dorsal side of right ear in mice once a day from days 0 to 6 to induce AD model, control mice were applied with 20 μL ethanol on the dorsal side of right ear as solvent control. MCC950 treatment mice was received MCC950 (10 mg/kg) intraperitoneally every two days, and VX-765 treatment group was administered intragastrically with VX-765 (50 mg/kg) daily. Control mice were treated with equal volume of saline. **B** Ear thickness (μm) of mice was measured daily in the same region of ear tissue using a vernier caliper. **C** Ear weight (mg) of mice was assessed 24 h after MC903 final application on day 7 in the same volume of ear tissue obtained by a tissue punch. **D** Ear histological changes were evaluated with H&E staining and images were observed with optical microscopy (magnification: ×200, scale bar: 100 μm). **E** Serum level of IgE was assessed by ELISA. **F** IL-33 secretion level was evaluated by ELISA from ear homogenates. **G** Immunoblot analysis of NLRP3 and IL-33 expression in ear homogenates with indicated treatments and the corresponding gray analysis from three mice. **H** qRT-PCR analysis of *Nlrp3* mRNA expression in ear homogenates derived from the same mice as applied in G. **I** Secretion level of IL-1β was evaluated by ELISA from ear homogenates. **J** Immunoblot analysis of NLRP3, caspase-1 p45, and caspase-1 p10 expression in LPS (10 μg/mL, 24 h) and ATP (5 mM, 1 h) stimulated HaCaT cells, or LPS (1 μg/mL, 6 h) treated Raw264.7 cells, respectively. Statistical comparisons were performed using One-way ANOVA analysis of variance with Dunnett’s test in multiple comparison, and unpaired two-tailed Student’s *t* test in two groups comparison (all data are represented as the mean ± SD, *n* = 6, *P < 0.05; **P < 0.01 vs. Control).

As noted above, IL-1β and IL-18 are the main terminal products generated by the activation of the inflammasome. However, we found the mRNA expression and cytokine secretion of IL-1β and IL-18 were not affected by NLRP3 in epithelium both in vivo and ex vivo. We then suspected whether NLRP3 could mediate IL-33 expression and secretion in epithelium independently of the inflammasome. We evaluated the expression and secretion of IL-33 in MC903-induced AD model, in which MCC950, a specific inhibitor of NLRP3 inflammasome activation and ASC oligomerization [30, 31], and VX-765, a selective inhibitor of caspase-1 activity [32] were applied (Fig. 3A). We found MCC950 or VX-765 application showed barely effect on the severity of epidermis inflammation in AD mice, including ear thickness (Fig. 3B), ear weight (Fig. 3C), histomorphology changes of ear epithelium (Fig. 3D), and serum level of IgE (Fig. 3E). Consistently, MCC950 did not result in an appreciable suppression of IL-33 production (Fig. 3F), as well as protein expression (Fig. 3G) in AD mice. Although MCC950 showed a mildly suppression of NLRP3 protein expression (Fig. 3G), no changes of *Nlrp3* mRNA expression in ear homogenates from AD lesions were detected (Fig. 3H). In addition, inhibition of caspase-1 activity with VX-765 had no effect on IL-33 secretion (Fig. 3F), and even induced a modest increase of IL-33 expression in AD mice (Fig. 3G). However, the secretion of IL-1β was not affected either in AD mice or mice that applied with MCC950 or VX-756 (Fig. 3I).

Activation of inflammasome can also be investigated by assessing the cleavage of caspase-1 (as cleave into its p10 isoform). We found caspase-1 protein indeed expressed in HaCaT cells, however, in contrast to NLRP3, the expression of caspase-1 p10 isoform was not detectable upon to stimulation (Fig. 3J). As a control, an increased caspase-1 p10 expression in Raw264.7 cells (murine macrophages) upon stimulation was observed (Fig. 3J). These results demonstrated that NLRP3 regulated IL-33 expression and release in the epithelium in an inflammasome-independent manner.

### IRF4 interacts with NLRP3 to control IL-33 expression in epithelial cells

**Fig. 4:**
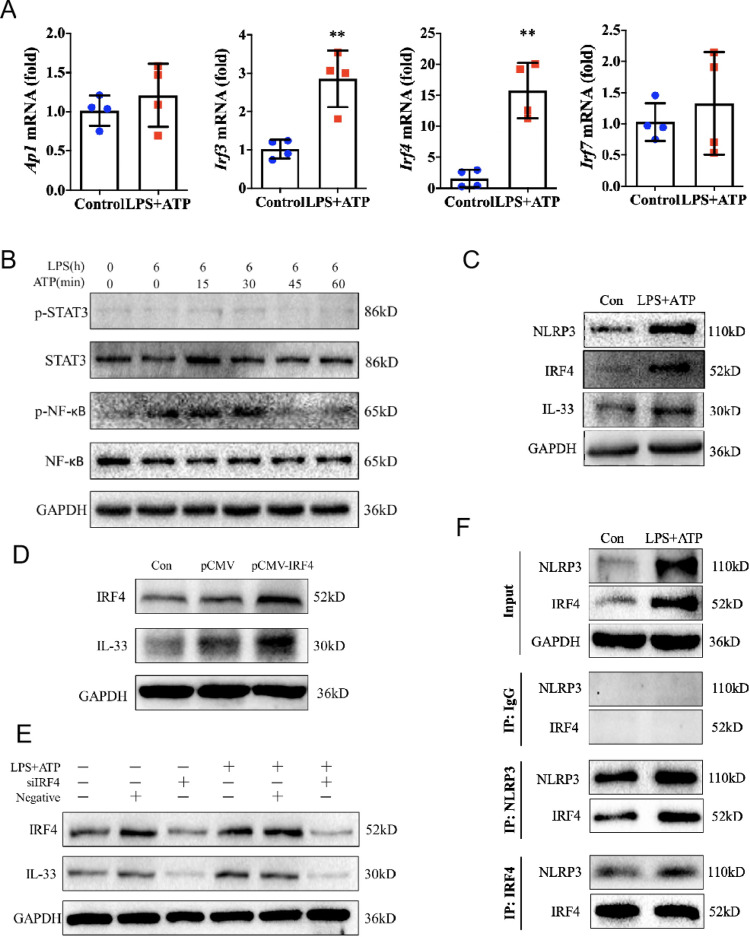
IRF4 interacts with NLRP3 to control IL-33 expression in epithelial cells. **A** Expression of *Ap1*, *Irf3*, *Irf4*, and *Irf7* mRNA in HaCaT cells after LPS (10 μg/mL, 6 h) and ATP (5 mM, 1 h) stimulation was evaluated by qRT-PCR. **B** Immunoblot analysis of p-STAT3, STAT3, p-NF-κB, and NF-κB in HaCaT cells after LPS (10 μg/mL, 6 h) and ATP (5 mM, with indicated time points) stimulation. **C** Immunoblot analysis of NLRP3, IRF4, and IL-33 expression in HaCaT cells in response to LPS (10 μg/mL, 24 h) and ATP (5 mM, 1 h) stimulation. **D–E**, Immunoblot analysis of IRF4 and IL-33 expression in HaCaT cells following overexpression of IRF4 by transfecting with pCMV-IRF4, or interfering of IRF4 with specific siRNA. **F** Precipitation of NLRP3 or IRF4 in HaCaT cells in response to LPS (10 μg/mL, 24 h) and ATP (5 mM, 1 h) stimulation following by immunoblot analysis of NLRP3 and IRF4 expression. Statistical comparisons were performed using unpaired two-tailed Student’s *t* test (all data are represented as the mean ± SD of three independent experiments, *n* = 4, *P < 0.05; **P < 0.01 vs. Control).

To better understand the underlying mechanism of NLRP3 in the regulation of IL-33 expression in epithelial cells, we performed the analysis of mRNA expression of *Ap1*, *Irf3*, *Irf4*, and *Irf7*, as well as phosphorylation activation of NF-κB and STAT3, the potential transcription factors known to be involved in the regulation of IL-33 expression in myeloid cells [33–35]. We found LPS combined with ATP stimulation did not raise mRNA expression of *Ap1* and *Irf7* in HaCaT cells, while *Irf4* mRNA expression increased significantly, and *Irf3* mRNA expression showed a modest increase (Fig. 4A). Moreover, the phosphorylation of STAT3 did not alter in response to stimulus, whereas the phosphorylation of NF-κB (p65) showed a transient increase, which might due to LPS is a TLR4 agonist (Fig. 4B). It has been proposed that IRF4 expression is confined to immune cells where it is a key factor in the regulation of cell differentiation [36]. The role of IRF4 in epithelial cells is not clear yet. We then speculated whether IRF4 was involved in NLRP3-mediated IL-33 expression in epithelial cells. We found IRF4 indeed expressed in the resting state of HaCaT cells (Fig. 4C). In line with increased expression of NLRP3 and IL-33 upon to stimulation, an upregulated expression of IRF4 was observed in HaCaT cells (Fig. 4C). Furthermore, overexpression of IRF4 in HaCaT cells substantially induced an enhanced expression of IL-33 (Fig. 4D), whereas siRNA-mediated silencing of the gene encoding IRF4 abolished the expression of IL-33 (Fig. 4E). These data suggested the transcription factor IRF4 was involved in NLRP3-mediated IL-33 expression in epithelial cells.

It seemed that both the NLRP3 and IRF4 were involved in IL-33 expression in epithelial cells, which led us to suspect the relationship between these two molecules. We noted a physical interaction between IRF4 and NLRP3 protein in the resting state of HaCaT cells, which was enhanced to a certain extent under LPS + ATP stimulation by immunoprecipitation assay (Fig. 4F). It indicated that NLRP3 and IRF4 might mediate IL-33 expression through an interaction in epithelial cells.

### NLRP3 is a transcription factor of IL-33 in epithelial cells

**Fig. 5:**
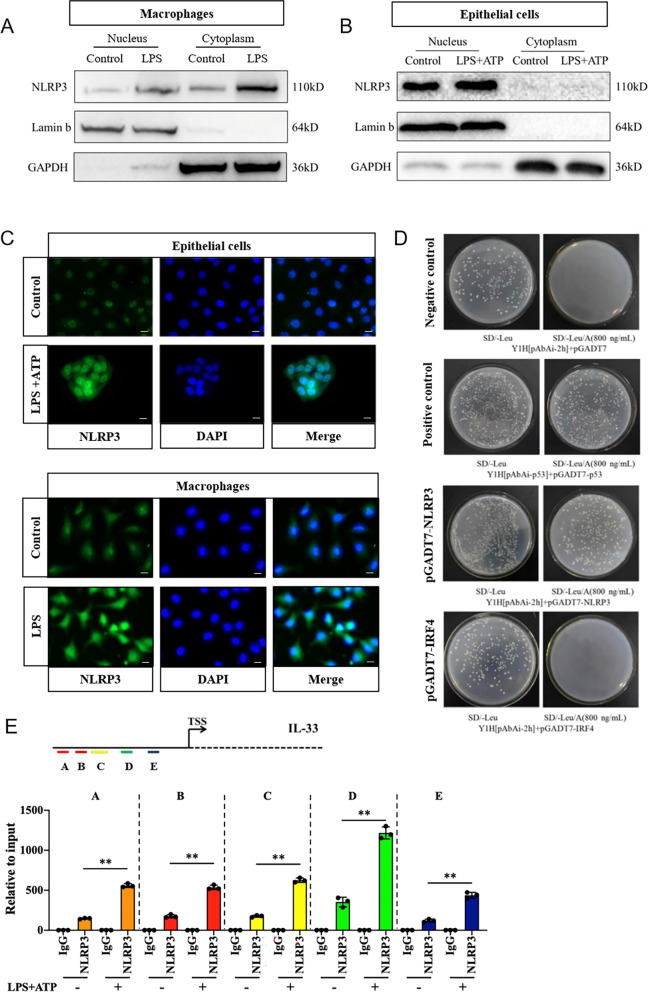
NLRP3 is a transcription factor of IL-33 in epithelial cells. **A** Immunoblot analysis of NLRP3 expression in the nucleus or cytoplasm from LPS (1 μg/mL, 6 h) stimulated Raw264.7 cells. **B** Immunoblot analysis of NLRP3 expression in nucleus or cytoplasm from LPS (10 μg/mL, 24 h) and ATP (5 mM, 1 h) activated HaCaT cells. **C** Immunofluorescence assay detection of NLRP3 localization in HaCaT cells in response to LPS (10 μg/mL, 24 h) and ATP (5 mM, 1 h) stimulation or in peritoneal macrophages in response to LPS (1 μg/mL, 6 h) stimulation (magnification: ×400, scale bar: 25 μm). **D** Yeast-one-hybrid (Y1H) assay analysis of the ability of NLRP3 or IRF4 to induce *Il33* transcription by using NLRP3 or IRF4 prey plasmids, and IL-33 DNA element bait plasmid with MATCHMAKER GAL4 one-hybrid system. **E** ChIP assay was performed in HaCaT cells with or without LPS (10 μg/mL, 24 h) and ATP (5 mM, 1 h) stimulation. Immunoprecipitation was performed using anti-NLRP3 antibody, and qRCR analysis conducted using five specific-promotor regions of *Il33* (**A, B, C, D**, and **E**). Statistical comparisons were performed using unpaired two-tailed Student’s *t* test (all data are represented as the mean ± SD, *n* = 3, *P < 0.05; **P < 0.01 vs. Control).

Our above findings suggested NLRP3 in epithelial cells was able to induce IL-33 expression independently of inflammasome and had an interaction with IRF4, which led us to hypothesize NLRP3 might act as a co-transcription factor for IRF4 to govern IL-33 expression in nucleus. We therefore addressed the protein localization of NLRP3 in epithelial cells. As a control, NLRP3 was found in both the cytoplasm and nucleus of macrophages, but mainly in the cytoplasm by immunoblot analysis (Fig. 5A), which was confirmed with immunofluorescence staining (Fig. 5C). In contrast, we found NLRP3 located predominantly in the nucleus of HaCaT cells (Fig. 5B), which was further confirmed by immunofluorescence observation (Fig. 5C). This implied that NLRP3 may function as a transcription factor in the nucleus of epithelial cells. To further document the role of NLRP3 and IRF4 in conducting *Il33* gene transcription, we generated NLRP3 and IRF4 prey plasmids and IL-33 DNA element bait plasmid to perform the yeast-one-hybrid (Y1H) assay. At the minimum concentration (800 ng/mL) of aureobasidin A (AbA) that could inhibit IL-33 self-transactivation (Supplementary Fig. 2), colony growth was observed in the plate with pGADT7-NLRP3 transfection, while no colony growth was observed in the plate with pGADT7-IRF4 transfection, indicating NLRP3, but not transcription factor IRF4, could directly bind to IL-33 DNA element and induce *Il33* gene expression (Fig. 5D). To further confirm the DNA-binding ability and transcriptional activity of NLRP3, we performed chromatin immunoprecipitation (ChIP) coupled to q-PCR. Results revealed that NLRP3 was able to bind to DNA fragments and further trigger *Il33* gene expression with five candidate promotors, and the binding ability was significantly upregulated in response to stimulation (Fig. 5E). Together these results documented that through an interaction with IRF4 in the nucleus, NLRP3 acted like a transcription factor in *Il33* gene expression in epithelial cells.

### NLRP3 deficiency impedes IL-33 expression and secretion in murine AD model

**Fig. 6:**
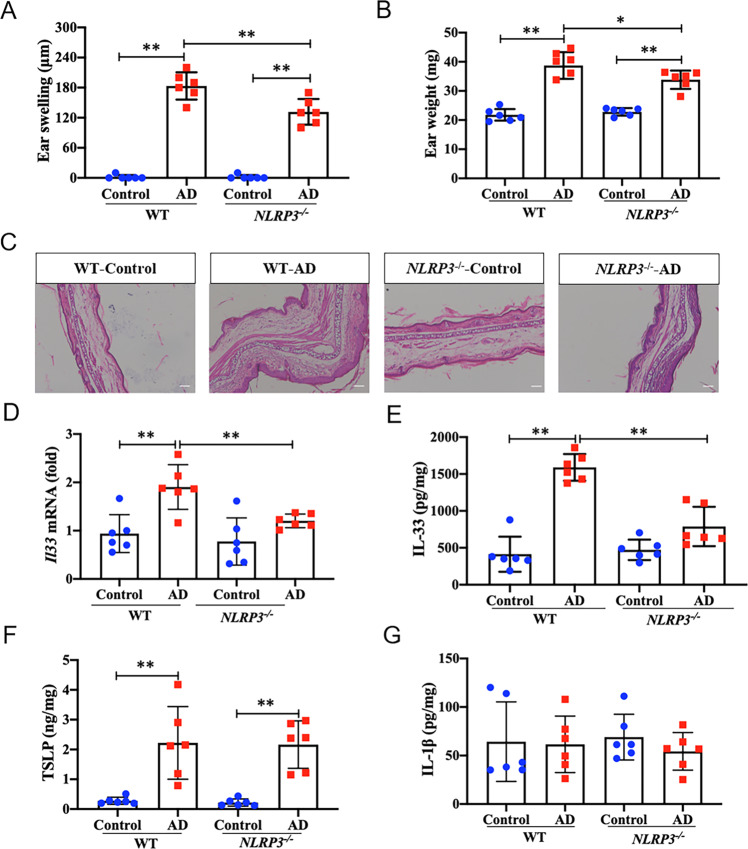
NLRP3 deficiency impedes IL-33 expression in atopic dermatitis model. **A** Ear thickness (μm) of mice was measured 24 h after MC903 final application on day 7 by using a vernier caliper from wild-type (WT) or *Nlrp3*^-/-^ mice. Ear swelling was obtained by analysis of the thickness difference between the left and right ears of each mouse. **B** Ear weight (mg) of mice was assessed 24 h after MC903 final application on day 7 in the same volume of ear tissue obtained by a tissue punch. **C** Ear histological changes were evaluated with H&E staining and images were observed with optical microscopy (magnification: ×100, scale bar: 100 μm). **D**, qRT-PCR analysis of *Il33* mRNA expression in ear homogenates from WT or *Nlrp3*^-/-^ mice. **E** Secretion level of IL-33 in ear homogenates from WT or *Nlrp3*^-/-^ mice was evaluated by ELISA. **F–G**, Secretion level of TSLP and IL-1β was evaluated by ELISA in ear homogenates from WT or *Nlrp3*^-/-^ mice. Statistical comparisons were performed using One-way ANOVA analysis of variance with Dunnett’s test in multiple comparison (all data are represented as the mean ± SD of two independent experiments, *n* = 6, *P < 0.05; **P < 0.01 vs. Control or WT-AD).

To further confirm the in vivo relevance of our observations, we used NLRP3 deficient (*NLRP3*^*-/-*^) mice or wild-type (WT) mice to evaluate the effect of NLRP3 on IL-33 mediated AD pathological process. After MC903 topically application for 7 days, AD models were successfully established in WT mice (Fig. 6A–C). In line with our hypothesis, we observed a significant reduced ear swelling and ear weight from MC903-treated *NLRP3*^*-/-*^ mice compared to that from WT mice (Fig. 6A, B). In addition, inflammatory infiltration in the morphology of AD lesions from *NLRP3*^*-/-*^-AD mice was alleviated relative to that in WT-AD mice (Fig. 6C). Consistently, *NLRP3*^*-/-*^-AD mice displayed notably decreased mRNA expression and secretion of IL-33 in ear homogenates of AD lesions relative to WT-AD mice (Fig. 6D, E). Nevertheless, as a control, no obvious changes were observed in terms of TSLP and IL-1β production in ear homogenates from both *NLRP3*^*-/-*^ or WT mice (Fig. 6F, G). Together these data confirmed that NLRP3 deficiency impeded IL-33 expression and production, and alleviated the epidermis inflammation of AD in vivo.

## Discussion

IL-33 is a proinflammatory cytokine that is abundant in the epidermal keratinocytes of patients with AD [37]. Overexpression of IL-33 in keratinocytes of mice led to AD-like immune pathology [2], suggesting the potent role of IL-33 in promoting AD. As a member of the IL-1 cytokine family, the expression and secretion of IL-33 were initially proposed that mediated by NLRP3 inflammasome [38]. However, the role of NLRP3 inflammasome in AD pathology is highly controversial. It has been shown that NLRP3 inflammasome signaling pathway was highly upregulated in the skin of mice with chronic dermatitis [39]. In contrast, a published study reported no role of NLRP3 inflammasome in the development of allergic inflammation in AD mice model [40]. In current study, based on the RNA sequencing results obtained from GEO dataset (GSE130588), we found both the *Il33* and *Nlrp3* mRNA expressions were increased in lesional skin of AD patients when compared with healthy subjects. To further study the role of IL-33 and NLRP3 in the pathogenesis of AD, we conducted AD-like skin inflammation in mice by topically applying with MC903. A 7-day time course induced mice ear skin thickening starting as early as day 2 and dramatically progressing up to day 7. Histologically, inflammatory cell infiltration and edema were observed in the lesional skin of AD mice. We also found the serum level of IgE and the skin expression of Type 2 and alarmin cytokines, such as IL-4, IL-33, and TSLP were typically elevated in MC903-induced AD mice. These results suggested that topically applying MC903 in mice could induce key features associated with AD. Consistent with GEO dataset analysis of AD patients, an obvious enhanced NLRP3 protein and mRNA expression in the skin epidermis of MC903-induced AD mice were observed. These results indicated that IL-33 and NLRP3 may play an important role in the pathogenesis of AD. Recent studies revealed that the cytokines IL-36α, IL-36β, and IL-36γ, which belong to the IL-1 family, possess proinflammatory and anti-inflammatory activities and may take part in the pathogenesis of allergy [41]. An increased expression of IL-36α and IL-36γ in the lesional skin of AD patients compared to non-lesional skin was reported, although the upregulation was unexpectedly small [29]. In contrast, we did not observe any significant changes in *Il36α* and *Il-36γ* mRNA expressions in MC903-mediated AD mice. Consistently, a study of IL-36 in inflammatory skin diseases has pointed out that the IL-36 cytokine family was identified as “psoriasis-specific”, when compared the mRNA profiles of biopsies from AD/psoriatic lesional and non-lesional skin [42]. In addition, it has been demonstrated that IL-36 may play an important role in AD pathogenesis especially upon *Staphylococcus aureus* colonization [43, 44], whereas we used MC903 in current study to initiate AD-like allergic inflammation. Correlation analysis implied the mRNA expression of *Nlrp3* was only positively correlated with *Il33*, but not *Tslp*, *Il4*, *Il36α*, *Il-36γ*, *Il1β*, and *Il18* mRNA expression in epithelium in vivo. In human immortilized epithelial cells, we confirmed NLRP3 was involved in IL-33 expression and secretion. We initially suspected the enhanced IL-33 expression and secretion in the epithelium of AD were due to NLRP3 dictated inflammasome activation. However, as the terminal products of inflammasome activation, IL-1β and IL-18 secretion levels showed barely changes in epithelium both in vivo and ex vivo. In addition, application with MCC950, a highly potent specific inhibitor of the NLRP3 inflammasome, which can block NLRP3-induced ASC oligomerization [30], showed no effect on epidermal inflammation and IL-33 secretion of mice mediated by MC903. Furthermore, the cleavage of caspase-1 into p10 isoform was not detectable in response to classic activators of NLRP3 in epithelial cells. In fact, the role of caspase-1 in processing and secretion of bioactive IL-33 is currently controversial. Similar to IL-1β and IL-18, cleavage of pro-IL-33 into mature IL-33 by caspase-1 was believed to be essential for optimal biologic activity. In contrast, it has been shown caspase-1 processing resulted in inactivation rather than activation of IL-33 [38]. Moreover, caspase-1-deficient macrophages were able to release IL-33 after LPS stimulation, which suggested inflammasome and caspase-1 are dispensable for IL-33 expression and secretion [45]. We observed that application with VX-765, an inhibitor of caspase-1 activity, did not alter IL-33 expression and secretion in AD mice model. Therefore, our results suggested the ability of NLRP3 expressed in epithelium to regulate IL-33 expression and secretion in the pathological process of AD might independently of inflammasome.

Several transcriptional regulators have been identified to mediate IL-33 expression, including STAT3 [33], NF-κB [46], AP-1 [47], IRF3 [48], IRF7 [34], and IRF4 [35]. In this report, we found STAT3, NF-κB, AP-1, and IRF7 might not be responsible for IL-33 transcription in HaCaT cells. However, we observed IRF4 and IRF3 were associated with IL-33 expression in epithelial cells, especially IRF4, as overexpression or interfering of IRF4 could enhance or abolish IL-33 expression. IRF4 is a member of the interferon regulatory factor family (IRF) of transcription factor. It has been proposed the DNA-binding ability of IRF4 is relatively weak, and it requires heterodimerization with other partners such as BATF or Jun to induce transcription [49]. It has been shown that NLRP3 is a potential partner of IRF4 required for optimal IRF4-mediated gene transactivation [26]. In this report, we detected an enhanced physical interaction between NLRP3 and IRF4 in LPS and ATP stimulated epithelial cells. In addition, we found NLRP3, but not IRF4, could directly bind to IL-33 DNA element and induce *Il33* gene expression by Y1H assay, which led us to suspect NLRP3 might be function as a transcription factor of IL-33 in epithelial cells.

The transcription functions of nucleotide-binding oligomerization domain-like receptor (NLR) family have been reported previously, such as the major histocompatibility complex class II transactivator CIITA [50], and NLRP5 [51, 52]. As another member of NLR family, we found NLRP3 was located predominantly in the nucleus of epithelial cells as opposed to macrophages, where it was found mainly in cytoplasm. The different subcellular localization of NLRP3 between the macrophages and epithelial cells may account for the different functions of NLRP3. In the nucleus of epithelial cells, we found NLRP3 could directly bind to *Il33* promoters and induce an upregulated transactivity, which demonstrated its ability to sense DNA as a transcription factor. These results highly implied the nuclear location might favor the transcriptional function of NLRP3.

We further confirmed the role of NLRP3 in the regulation of IL-33 expression and secretion in the pathological process of AD in *Nlrp3*^*-/-*^ mice. We observed the allergic epidermis inflammation as well as IL-33 secretion was impeded in *Nlrp3*^*-/-*^ mice relative to WT mice. However, no changes were detected in terms of TSLP and IL-1β production either in *NLRP3*^*-/-*^ or in WT mice. We also noted the pathological changes of AD were not completely rescued in *Nlrp3*^*-/-*^ mice. It is probably due to other alarmins or proinflammatory cytokines that involved in pathology of AD, such as TSLP, IL-4, IL-13 etc., might not be directly regulated by NLRP3, as we did not observe TSLP production was affected in *Nlrp3*^*-/-*^-AD mice.

Together our results have demonstrated the transcriptional function of NLRP3 independently of inflammasome and that it was able to interact with IRF4 and bind with DNA to govern IL-33 expression and secretion in epithelial cells. In addition, our results showed that NLRP3-mediated IL-33 expression and secretion had a physiological relevance in the allergic inflammation of AD, which may contribute to a better understanding of the role of NLRP3 and IL-33 in the pathogensis of AD. Furthermore, our work provides the impetus to generate agents that targeting the transcription function of NLRP3 in epithelial cells might be the development of antiallergic disease drugs.

## Materials and methods

### Mice

Male BALB/c were purchased from Beijing Vital River Laboratory Animal Technology Co., Ltd. C57BL/6 (WT) and NLRP3 deficient (*Nlrp3*^-/-^) mice were presented as a gift from Prof. Rongbin Zhou (University of Science and Technology of China, China). All animal were raised at Nanjing University of Chinese Medicine under specific pathogen-free condition at 18–25 ^o^C and 50–60% humidity, and were used at 6–8 weeks of age. Mice were allocated to experimental groups with weight-stratified randomization. The investigators were blinded to the group allocation during the experiments. All procedures involving animals were approved by the Animal Care and Ethical Committee of Nanjing University of Chinese Medicine and were performed strictly according to the Guide for the Care and Use of Laboratory Animals.

### Reagents

MC903 was purchased from MCE (HY-10001, China). MCC950 (CP-456773) and VX-765 (S2228) were obtained from Selleck (China). LPS and ATP were obtained from Sigma-Aldrich and Biosharp Life Science (China), respectively.

### MC903 model of atopic dermatitis and in vivo treatments

To establish AD-like allergic inflammation in mice, MC903 was applied according to the published protocol [28]. Briefly, MC903 (2 nM dissolved in 20 μL ethanol) was topically applied to dorsal side of right ear in BALB/c mice once a day from days 0 to 6, control mice were applied with 20 μL ethanol on the dorsal side of right ear (*n* = 5). To evaluate the effects of NLRP3 inflammasome signaling on MC903-induced AD, MCC950 (10 mg/kg) or VX-765 (50 mg/kg) was administered to MC903-applied BALB/c mice intraperitoneally every two days or intragastrically daily, respectively, control and AD mice were treated with equal volume of saline (*n* = 6). To validate the effect of NLRP3 on IL-33 processing, MC903-mediated AD model was established in C57BL/6 (WT) and *Nlrp3*^-/-^ mice as described above (*n* = 6). Ear thickness of mice was assessed daily using a vernier caliper (Mitutoyo, Japan). At 24 h after MC903 final application, peripheral blood of mice was collected from orbit, and mice were scarified to collect ear tissues either stored at -80 ^o^C or fixed overnight in buffered 4% formaldehyde solution.

### Cell culture

HaCaT (ID: 3111C0001CCC000373) and Raw264.7 cells (ID: 3111C0001CCC000146) were purchased from the Cell Resource Center, Peking Union Medical College (which is the headquarter of National Infrastructure of Cell Line Resource, NSTI, Beijing, China). Cells were identified with short tandem repeat profiling and tested for mycoplasma contamination. HaCaT cells (2 × 10^5^/well) were cultured with MEM, and Raw264.7 cells (2 × 10^5^/well) were maintained in DMEM, supplemented with 10% FBS (Hyclone Laboratories, Inc.) in a 5% CO_2_ incubator at 37 ^o^C. For cell stimulation, HaCaT cells were pretreated with LPS (10 μg/mL) for 6 h following with addition of ATP (5 mM) for 1 h or indicated time points, Raw264.7 cells were treated with LPS (1 μg/mL) for 6 h.

### Histology and immunohistochemistry

Histopathology observation was performed as described previously [53]. Expression of NLRP3 and IL-33 in ear tissues were assessed by immunohistochemistry (IHC) staining using NLRP3 (Bioss, BS-10021R) and IL-33 (Enzo Life Sciences, ALX-804–840-C100) antibodies, and observed with optical microscopy (Axion A1; Carl Zeiss AG, Germany).

### ELISA

Cell culture supernatants, ear homogenates or serum samples were assayed by ELISA with human IL-1β (DKW12-1012-096, Dakewe, China), human IL-18 (EK1180, MultiSciences, China), human IL-33 (BMS2048, Invitrogen), mouse IL-1β (EK201B, MultiSciences, China), mouse IL-18 (EK218–96, MultiSciences), mouse IL-33 (88-7333-88, Invitrogen), mouse TSLP (88-7490-88, Invitrogen), or mouse IgE (88-50460-88, Invitrogen) kits according to the manufactures’ instructions.

### Immunoblot analysis

Protein extracts were prepared by lysing of ear homogenates or cells for 30 min at 4 ^o^C in RIPA buffer (P0013C, Beyotime Biotechnology, China) in the presence of PMSF (BL507A, Biosharp Life Science, China). For nuclear and cytoplasmic protein extraction, a pellet of 1 × 10^7^ cells were extracted by a Nuclear and Cytoplasmic Protein Extraction Kit (P0027, Beyotime Biotechnology, China). Protein concentrations were detected with a BCA kit (P0011, Beyotime Biotechnology, China). Protein lysates were then subjected to 10% SDS-PAGE and with the use of antibodies (Supplementary Table 1) following with horseradish peroxidase-electrochemiluminescence detection (OR03L, Millipore). The relative intensities of protein blots were analyzed ChemiScope analysis software.

### RNA extraction and quantitative real-time PCR

Total RNA was extracted from cells or ear tissues using an RNA isolator Total RNA Extraction Reagent (R401-01, Vazyme Biotech Co., Ltd). The cDNA was prepared using HiScript® II Q Select RT SuperMix (R233-01, Vazyme Biotech Co., Ltd). Gene-specific real-time quantitative PCR was performed using primers (Supplementary Table 2, ordered from Genscript (China)) with AceQ qPCR SYBR Green Master Mix (Q111-02, Vazyme Biotech Co., Ltd). The results for each gene were normalized to that for GAPDH expression in the samples and analyzed using ΔΔCt method.

### siRNA interference

HaCaT cells were transiently transfected with specific siRNA using Lipofectamine™ 2000 Transfection Reagent (11668019, ThermoFisher). Cells were then recovered for 24 h before stimulation. The siRNA oligonucleotides (Supplementary Table 3) were designed and synthesized by Hanbio Biotechnology (China).

### Overexpression of NLRP3 or IRF4 in HaCaT cells

Overexpression of NLRP3 or IRF4 in HaCaT cells were performed by transfecting with pLV-EGFP-NLRP3 (NM_001079821.1, Cyagen Biosciences, China) and pCMV-His-IRF4 (NM_002460.4, Hanbio Biotechnology, China) using Lipofectamine™ 2000 Transfection Reagent (11668019, ThermoFisher) according to the manufacturer’s instructions.

### Immunoprecipitation assay

Immunoprecipitation assay was performed with at least 1 × 10^7^ cells as described in published paper [54]. For immunoprecipitation, cells were lysed in RIPA buffer (P0013C, Beyotime Biotechnology, China) and precleared with Protein A/G Agarose beads (36403ES05, Yeasen Biotechnology, China). Pull-down antibodies and Protein A/G Agarose beads were added and further incubated at 4 ^o^C for 16 h. Protein A/G Agarose beads were collected, samples were resolved on 10% SDS-PAGE, and analyzed for anti-NLRP3 and anti-IRF4 antibodies (Supplementary Table 1).

### Immunofluorescence

Subcellular protein localization was detected by immunofluorescent staining with the use of antibodies against NLRP3 (BS-20021R, Bioss) and FITC-conjugated IgG antibody (ab6717, Abcam). Nuclei were stained with DAPI (C1002, Beyotime). Staining was assessed with a live cell work station microscope (Carl Zeiss, Germany) with the use of identical settings between conditions.

### Chromatin immunoprecipitation (ChIP) assay

ChIP assay was performed using a MAGnify™ Chromatin Immunoprecipitation System (492024, ThermoFisher) according to the manufacturer’s instruction. At least 1 × 10^7^ cells were fixed with 1% formaldehyde for cross-linking of DNA with proteins, then cells were lysed to aid in nuclei release. DNA fragments by ultrasound were immunoprecipitated overnight at 4 ^o^C with 5 μg anti-NLRP3 antibody (Supplementary Table 1), or 5 μg of negative control immunoglobulin. After addition of protein magnetic beads, the mixture of protein magnetic beads, antibody and chromatin was washed and eluted from the magnetic beads with elution buffers. Then, cross-linking was reversed and samples were analyzed by quantitative PCR. The primer sequences were shown in Supplementary Table 4. Data were presented as the amount of DNA recovered relative to the input control.

### Yeast-one hybrid

The MATCHMAKER GAL4 one-hybrid system was employed according to the manufacturer’s instructors (Clontech). The full-length sequences of NLRP3, IRF4 (Supplementary Table 5) were separately cloned into the pGADT7 vector. The promoter sequence of IL-33 was cloned into the pAbAi vector. Empty pGADT7 vector was used as a negative control, and the interaction between pGADT7-p53 and pAbAi-p53 served as a positive control. These constructs were transformed into the yeast strain AH109 and subjected to selection on SD/-Leu/AbA medium plates at 30 ^o^C for 3–5 days.

### Statistical analysis

All data were expressed as the mean ± SD. Unpaired two-tailed Student’s *t* test was used when comparing two groups. One-way ANOVA analysis of variance with Dunnett’s test was applied to compare multiple groups. Statistical analysis was performed using GraphPad Prism 8 Software. All experiments were repeated at least three times. A *P* value of <0.05 was considered statistically significant for all experiments.

### Supplementary information

Supplementary Tables of reagents’ information

Supplementary Figures

Supplementary Figure legends

## Data Availability

The datasets used and/or analyzed during the current study are available from the corresponding author on reasonable request.
