# Correction: A novel function of NLRP3 independent of inflammasome as a key transcription factor of IL-33 in epithelial cells of atopic dermatitis

**DOI:** 10.1038/s41419-024-06605-w

**Published:** 2024-03-21

**Authors:** Jie Zheng, Lu Yao, Yijing Zhou, Xiaoqun Gu, Can Wang, Kaifan Bao, Yang Sun, Min Hong

**Affiliations:** 1 Jiangsu Key Laboratory for Pharmacology and Safety Evaluation of Chinese Materia Medica, School of Pharmacy, Nanjing University of Chinese Medicine, 138 Xianlin Avenue, Nanjing, 210023 China; 2 Department of Pharmacology, School of Medicine & Holistic Integrative Medicine, Nanjing University of Chinese Medicine, 138 Xianlin Avenue, Nanjing, 210023 China; 3 Department of Biotherapy, Nanjing Jinling Hospital, Nanjing, 210002 China; 4 Department of Immunology, School of Medicine & Holistic Integrative Medicine, Nanjing University of Chinese Medicine, 138 Xianlin Avenue, Nanjing, 210023 China; 5 State Key Laboratory of Pharmaceutical Biotechnology, Chemistry and Biomedicine Innovation Center, School of Life Sciences, Nanjing University, 163 Xianlin Avenue, Nanjing, 210023 China

**Keywords:** Mechanisms of disease, Inflammation

Correction to: *Cell Death and Disease* 10.1038/s41419-021-04159-9, published online 24 September 2021

We have noted that there was an error in Fig. 4C, in that the IRF4 for combination treatment with LPS and ATP in HaCaT cells by mistake duplicated for the input IRF4 in Fig. 4F with the exactly same stimulation condition.
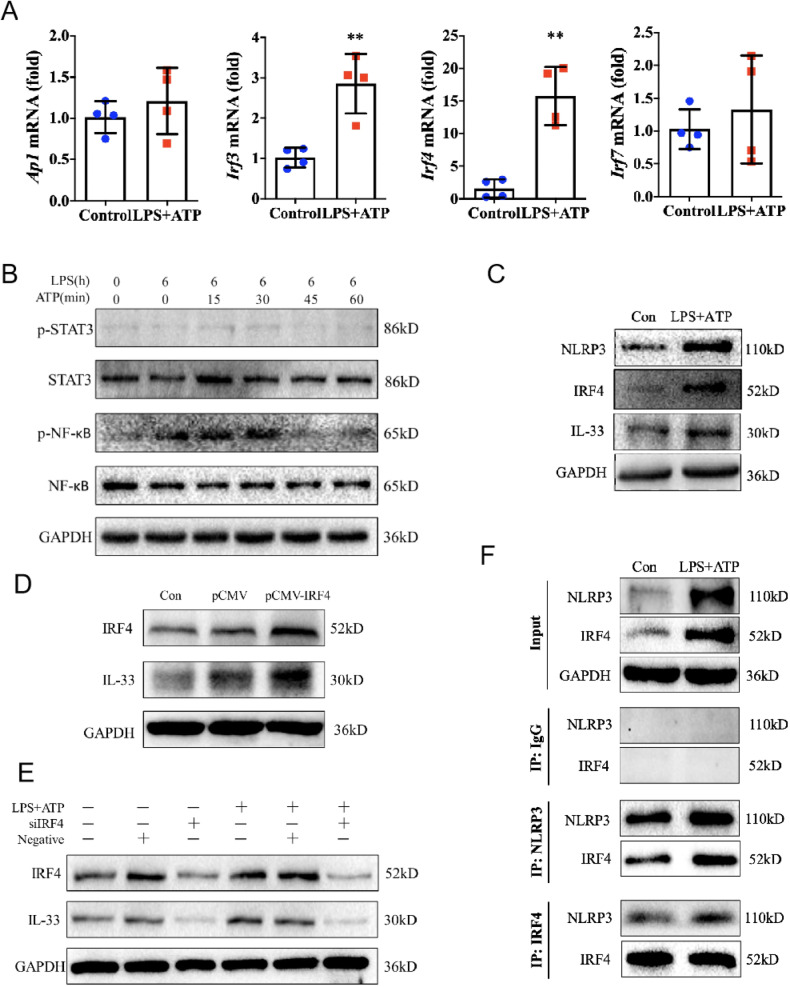

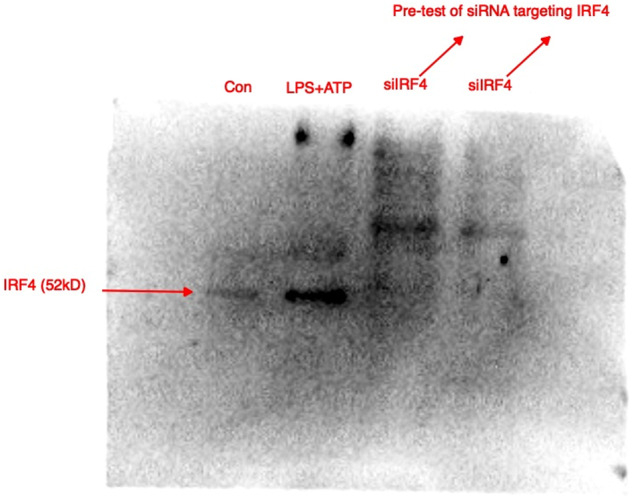

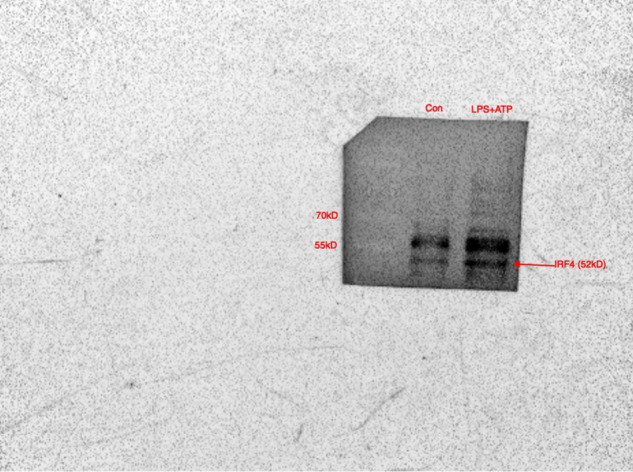

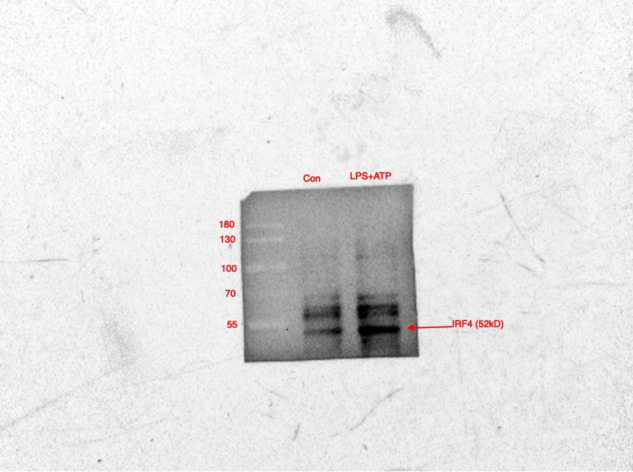


The original article has been corrected.

