# Supplementary material for: A novel function of NLRP3 independent of inflammasome as a key transcription factor of IL-33 in epithelial cells of atopic dermatitis
Source: Cell Death Dis. 2021 Sep 24;12(10):871. doi: 10.1038/s41419-021-04159-9 (PMC8463682; doi:10.1038/s41419-021-04159-9)
Supplement: Supplementary file 1 — Supplementary Tables of reagents’ information [file 41419_2021_4159_MOESM1_ESM.docx]

**Supplementary Tables of reagents’ information**

**Table 1. Antibodies used for immunoblot analysis**

| Antibodies | Cat No. | Company |
| --- | --- | --- |
| Anti-IL-33 | ab54385 | Abcam |
| Anti-caspase-1 | ab1872 | Abcam |
| Anti-IRF4 | ab133590 | Abcam |
| Anti-NLRP3 | AG-20B-0014-C100 | AdipoGen Life Sciences |
| Anti-STAT3 | sc-8019 | Santa Cruz Biotechnology |
| Anti-pSTAT3 | sc-8059 | Santa Cruz Biotechnology |
| Anti-NF-κB p65 | sc-8008 | Santa Cruz Biotechnology |
| Anti-pNF-κB p65 | 93H1 | Cell Signaling Technology |
| Anti-GAPDH | AB0036 | Abways |
| Anti-Lamin b | ab133741 | Abcam |

**Table 2. Primers used for quantitative real-time PCR**

| mRNA | Primers |
| --- | --- |
| Human NLRP3 forward | 5’-AAGGGCCATGGACTATTTCC-3’ |
| Human NLRP3 reverse | 5’-GACTCCACCCGATGACAGTT-3’ |
| Human IL-33 forward | 5’-CGGTGTTGATGGTAAGATG-3’ |
| Human IL-33 reverse | 5’-AGAGTGTTCCTTGTTGTTG-3’ |
| Human IL-1β forward | 5’-AGGATATGGAGCAACAAGT-3’ |
| Human IL-1β reverse | 5’-GCAGGACAGGTACAGATT-3’ |
| Human IL-18 forward | 5’-GATATGACTGATTCTGACTGTAG-3’ |
| Human IL-18 reverse | 5’-TACCTCTAGGCTGGCTAT-3’ |
| Human AP-1 forward | 5’-CCAACTCATGCTAACGCAGC-3’ |
| Human AP-1 reverse | 5’-TCTCTCCGTCGCAACTTGTC-3’ |
| Human IRF3 forward | 5’-TGGGCCCCCAGATCTGATTA-3’ |
| Human IRF3 reverse | 5’-CACACAGAACCAGAGGGCAT-3’ |
| Human IRF4 forward | 5’-TTTATGCTTGTGCCCCACCT-3’ |
| Human IRF4 reverse | 5’-TCGGCAGACCTTATGCTTGG-3’ |
| Human IRF7 forward | 5’-GAAGCGCCACTGTTTAGGTTTC-3’ |
| Human IRF7 reverse | 5’-TACCTCTAGGCTGGCTAT-3’ |
| Human GAPDH forward | 5’-GGTTGTCTCCTGCGACTTCA-3’ |
| Human GAPDH reverse | 5’-TGGTCCAGGGTTTCTTACTCC-3’ |
| Mouse NLRP3 forward | 5’-TCCTGGTGACTTTGTATATGCGT-3’ |
| Mouse NLRP3 reverse | 5’-TTCTCGGGCGGGTAATCTTC-3’ |
| Mouse IL-33 forward | 5’-TCACTGCAGGAAAGTACAGCAT-3’ |
| Mouse IL-33 reverse | 5’-TTTGCCGGGGAAATCTTGGA-3’ |
| Mouse IL-1β forward | 5’-TGCCACCTTTTGACAGTGATG-3’ |
| Mouse IL-1β reverse | 5’-AAGGTCCACGGGAAAGACAC-3’ |
| Mouse IL-18 forward | 5’-ACTCTTGCGTCAACTTCA-3’ |
| Mouse IL-18 reverse | 5’-CTGATTCCAGGTCTCCATT-3’ |
| Mouse IL-4 forward | 5’-GCTTGAAGAAGAACTCTAGTG-3’ |
| Mouse IL-4 reverse | 5’-GATGTGGACTTGGACTCA-3’ |
| Mouse TSLP forward | 5’-TACTATACTCTCAATCCTATCCCTG-3’ |
| Mouse TSLP reverse | 5’-ACTTCTTGTGCCATTTCCTG-3’ |
| Mouse IL-36α forward | 5’-TGCAGATTGGCAGCTCAGAA-3’ |
| Mouse IL-36α reverse | 5’-GGGAGCAAGGTAATAGTGACTGG-3’ |
| Mouse IL-36γ forward | 5’-GTTCCACGAAGCCACAGAGTAACC-3’ |
| Mouse IL-36γ reverse | 5’-TGGCAATCCCTTTGTCCTGTTCAAG-3’ |
| Mouse GAPDH forward | 5’-TCCTGGTGACTTTGTATATGCGT-3’ |
| Mouse GAPDH reverse | 5’-TTCTCGGGCGGGTAATCTTC-3’ |

**Table 3. siRNA sequences used for RNA interference**

| siRNA | Sequences |
| --- | --- |
| NLRP3 siRNA | 5’-CGCUAAUGAUCGACUUCAAUG-3’ |
| IL-33 siRNA | 5’-GGUAAGAUGUUAAUGGUAACC-3’ |
| IRF4 siRNA | 5’ -GAAGAUUACCACAGAUCUAUC-3’ |

**Table 4. Primers used for ChIP assay**

| IL-33 promotors | Sequences |
| --- | --- |
| NM_001199640.1-1 (IL-33 A) forward | 5’-CAAGCCTGGTCAGCCATCTGTT-3’ |
| NM_001199640.1-1 (IL-33 A) reverse | 5’-GTTCTGGAATCCAGGGGAAAGAG-3’ |
| NM_001199640.1-2 (IL-33 B) forward | 5’-CATCCTGTCCGCTTGCTTCACT-3’ |
| NM_001199640.1-2 (IL-33 B) reverse | 5’-GATGGCTATGTCTGCCCTTTGG-3’ |
| NM_001199640.1-3 (IL-33 C) forward | 5’-TTCCCTTGGCTGTTACTTGTCA-3’ |
| NM_001199640.1-3 (IL-33 C) reverse | 5’-GAGCTGTGTGCAATAAATAGGAGG-3’ |
| NM_001199640.1-4 (IL-33 D) forward | 5’-GGAGGGAGGACGCAGAAAGTAG-3’ |
| NM_001199640.1-4 (IL-33 D) reverse | 5’-CTGGGCGGGTGAGATTTTAGC-3’ |
| NM_001199640.1-5 (IL-33 E) forward | 5’-CAGCAGCAGTCTTCCTTCCAAT-3’ |
| NM_001199640.1-5 (IL-33 E) reverse | 5’-ACTTGGACTGATTCCACATCCTAAG-3’ |

**Table 5. The full-length sequences of proteins applied for Yeast-one hybrid assay**

| **Proteins** | **Full-length sequences** | |
| --- | --- | --- |
| **NLRP3** | | MASTRCKLARYLEDLEDVDLKKFKMHLEDYPPQKGCIPLPRGQTEKADHVDLATLMIDFNGEEKAWAMAVWIFAAINRRDLYEKAKRDEPKWGSDNARVSNPTVICQEDSIEEEWMGLLEYLSRISICKMKKDYRKKYRKYVRSRFQCIEDRNARLGESVSLNKRYTRLRLIKEHRSQQEREQELLAIGKTKTCESPVSPIKMELLFDPDDEHSEPVHTVVFQGAAGIGKTILARKMMLDWASGTLYQDRFDYLFYIHCREVSLVTQRSLGDLIMSCCPDPNPPIHKIVRKPSRILFLMDGFDELQGAFDEHIGPLCTDWQKAERGDILLSSLIRKKLLPEASLLITTRPVALEKLQHLLDHPRHVEILGFSEAKRKEYFFKYFSDEAQARAAFSLIQENEVLFTMCFIPLVCWIVCTGLKQQMESGKSLAQTSKTTTAVYVFFLSSLLQPRGGSQEHGLCAHLWGLCSLAADGIWNQKILFEESDLRNHGLQKADVSAFLRMNLFQKEVDCEKFYSFIHMTFQEFFAAMYYLLEEEKEGRTNVPGSRLKLPSRDVTVLLENYGKFEKGYLIFVVRFLFGLVNQERTSYLEKKLSCKISQQIRLELLKWIEVKAKAKKLQIQPSQLELFYCLYEMQEEDFVQRAMDYFPKIEINLSTRMDHMVSSFCIENCHRVESLSLGFLHNMPKEEEEEEKEGRHLDMVQCVLPSSSHAACSHGLVNSHLTSSFCRGLFSVLSTSQSLTELDLSDNSLGDPGMRVLCETLQHPGCNIRRLWLGRCGLSHECCFDISLVLSSNQKLVELDLSDNALGDFGIRLLCVGLKHLLCNLKKLWLVSCCLTSACCQDLASVLSTSHSLTRLYVGENALGDSGVAILCEKAKNPQCNLQKLGLVNSGLTSVCCSALSSVLSTNQNLTHLYLRGNTLGDKGIKLLCEGLLHPDCKLQVLELDNCNLTSHCCWDLSTLLTSSQSLRKLSLGNNDLGDLGVMMFCEVLKQQSCLLQNLGLSEMYFNYETKSALETLQEEKPELTVVFEPSW |
| **IRF4** | | MNLEGGGRGGEFGMSAVSCGNGKLRQWLIDQIDSGKYPGLVWENEEKSIFRIPWKHAGKQDYNREEDAALFKAWALFKGKFREGIDKPDPPTWKTRLRCALNKSNDFEELVERSQLDISDPYKVYRIVPEGAKKGAKQLTLEDPQMSMSHPYTMTTPYPSLPAQVHNYMMPPLDRSWRDYVPDQPHPEIPYQCPMTFGPRGHHWQGPACENGCQVTGTFYACAPPESQAPGVPTEPSIRSAEALAFSDCRLHICLYYREILVKELTTSSPEGCRISHGHTYDASNLDQVLFPYPEDNGQRKNIEKLLSHLERGVVLWMAPDGLYAKRLCQSRIYWDGPLALCNDRPNKLERDQTCKLFDTQQFLSELQAFAHHGRSLPRFQVTLCFGEEFPDPQRQRKLITAHVEPLLARQLYYFAQQNSGHFLRGYDLPEHISNPEDYHRSIRHSSIQE |
