# Supplementary figures and images for: A novel function of NLRP3 independent of inflammasome as a key transcription factor of IL-33 in epithelial cells of atopic dermatitis
Source: Cell Death Dis. 2021 Sep 24;12(10):871. doi: 10.1038/s41419-021-04159-9 (PMC8463682; doi:10.1038/s41419-021-04159-9)

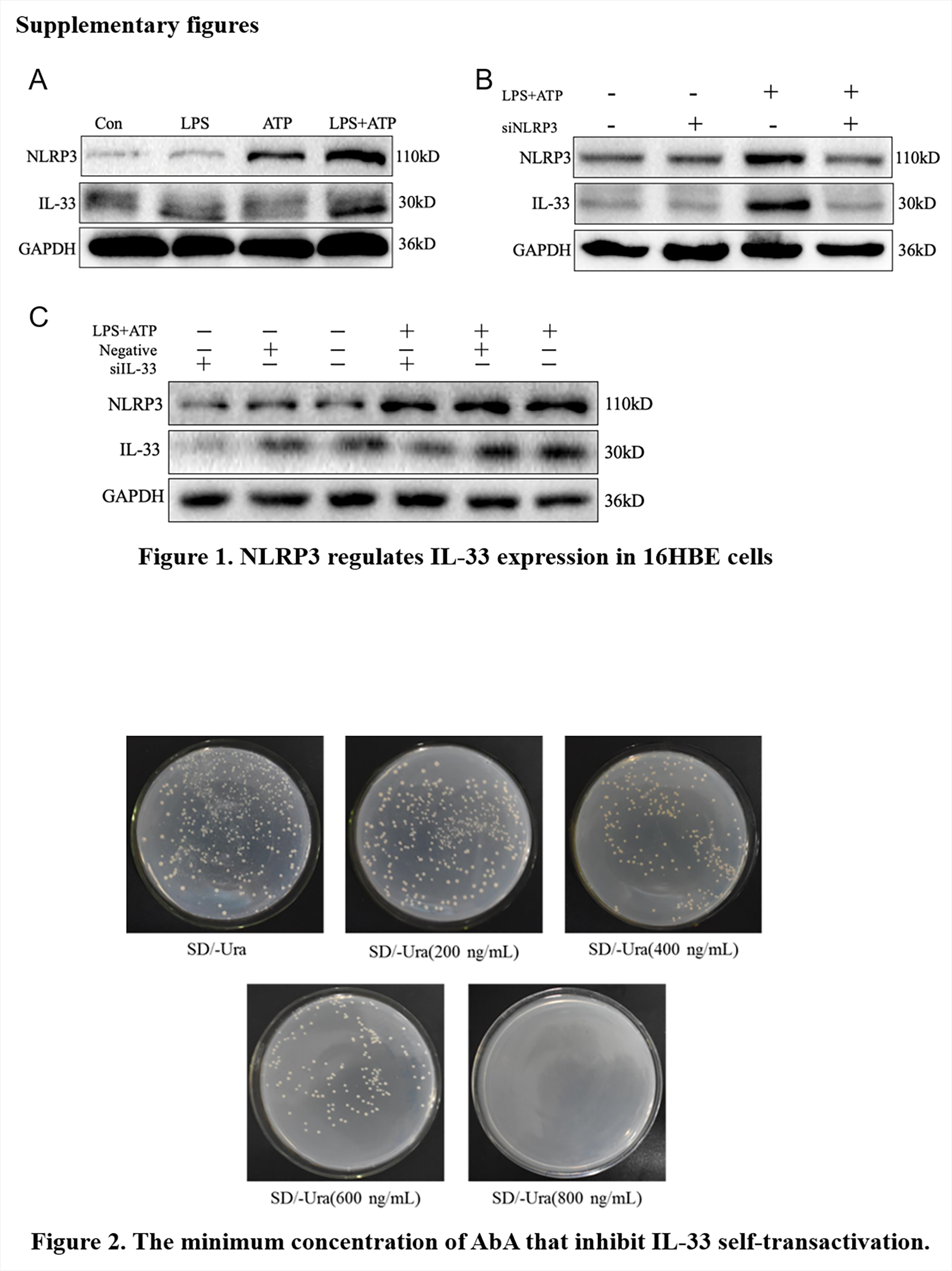

Supplement: Supplementary file 2 — Supplementary Figures [file 41419_2021_4159_MOESM2_ESM.tif]
