# Supplementary material for: A novel function of NLRP3 independent of inflammasome as a key transcription factor of IL-33 in epithelial cells of atopic dermatitis
Source: Cell Death Dis. 2021 Sep 24;12(10):871. doi: 10.1038/s41419-021-04159-9 (PMC8463682; doi:10.1038/s41419-021-04159-9)
Supplement: Supplementary file 3 — Supplementary Figure legends [file 41419_2021_4159_MOESM3_ESM.docx]

**Supplementary Figure legends**

**Figure 1. NLRP3 regulates IL-33 expression in 16HBE cells**

**A**, Immunoblot analysis of NLRP3 and IL-33 expression in 16HBE cells in response to LPS (10 μg/mL, 24 h), ATP (5 mM, 1 h), or LPS (10 μg/mL, 24 h) and ATP (5 mM, 1 h) stimulation. **B-C**, Immunoblot analysis of NLRP3 and IL-33 protein expression in 16HBE cells following interfering of NLRP3 or IL-33 with specific siRNAs.

**Figure 2. The minimum concentration of AbA that inhibit IL-33 self-transactivation.**

IL-33 bait plasmid self-activation was detected by Yeast one-hybrid (Y1H) analysis and the minimum ABA inhibitory concentration was 800 ng/mL.
